# A Deep Transfer Learning Framework for Sleep Stage Classification with Single-Channel EEG Signals

**DOI:** 10.3390/s22228826

**Published:** 2022-11-15

**Authors:** Hisham ElMoaqet, Mohammad Eid, Mutaz Ryalat, Thomas Penzel

**Affiliations:** 1 Department of Mechatronics Engineering, German Jordanian University, Amman 11180, Jordan; 2 Department of Biomedical Engineering, German Jordanian University, Amman 11180, Jordan; 3 Interdisciplinary Center of Sleep Medicine, Charité-Universitätsmedizin Berlin, 10117 Berlin, Germany

**Keywords:** sleep stage scoring, deep learning, recurrent neural network, long short-term memory, convolutional neural network, transfer learning

## Abstract

The polysomnogram (PSG) is the gold standard for evaluating sleep quality and disorders. Attempts to automate this process have been hampered by the complexity of the PSG signals and heterogeneity among subjects and recording hardwares. Most of the existing methods for automatic sleep stage scoring rely on hand-engineered features that require prior knowledge of sleep analysis. This paper presents an end-to-end deep transfer learning framework for automatic feature extraction and sleep stage scoring based on a single-channel EEG. The proposed framework was evaluated over the three primary signals recommended by the American Academy of Sleep Medicine (C4-M1, F4-M1, O2-M1) from two data sets that have different properties and are recorded with different hardware. Different Time–Frequency (TF) imaging approaches were evaluated to generate TF representations for the 30 s EEG sleep epochs, eliminating the need for complex EEG signal pre-processing or manual feature extraction. Several training and detection scenarios were investigated using transfer learning of convolutional neural networks (CNN) and combined with recurrent neural networks. Generating TF images from continuous wavelet transform along with a deep transfer architecture composed of a pre-trained GoogLeNet CNN followed by a bidirectional long short-term memory (BiLSTM) network showed the best scoring performance among all tested scenarios. Using 20-fold cross-validation applied on the C4-M1 channel, the proposed framework achieved an average per-class accuracy of 91.2%, sensitivity of 77%, specificity of 94.1%, and precision of 75.9%. Our results demonstrate that without changing the model architecture and the training algorithm, our model could be applied to different single-channel EEGs from different data sets. Most importantly, the proposed system receives a single EEG epoch as an input at a time and produces a single corresponding output label, making it suitable for real time monitoring outside sleep labs as well as to help sleep lab specialists arrive at a more accurate diagnoses.

## 1. Introduction

Sleep is central to human health and a healthy lifestyle. The health consequences of insufficient sleep, abnormal sleep patterns or de-synchronized circadian rhythms can be emotional, cognitive, or somatic. The disruption of normal sleep patterns has also been linked to obesity and neuro-degenerative diseases, as well as cardiac diseases [1,2]. The central diagnostic tool and the gold standard in the evaluation of sleep quality and disorders is the polysomnogram (PSG) or the overnight sleep study, during which several physiological signals are simultaneously monitored and collected, including electroencephalogram (EEG), electrooculography (EOG), electromyography (EMG), electrocardiography (ECG), blood oxygenation, airflow, and respiratory effort [3].

As defined by the American Academy of Sleep Medicine (AASM) [3], sleep is categorized into four stages. These stages include the stage of Rapid Eye Movement (REM) sleep and three non-REM (NREM) stages (N1, N2, and N3). A Wake (W) stage is also added to these stages, defining the class of awakening of the subject before or interrupting the sleep [4]. Usually, each sleep cycle goes through the non-REM stages’ (N1, N2, and N3) sleep to REM sleep. In most cases, the cycle takes 90–120 min, resulting in four to five cycles per night [5]. The duration of NREM stages is longer in early cycles of sleep, whereas the period of REM stage increases in later cycles. The electrical activity captured by sensors positioned throughout the body during the sleep study is what determines these sleep stages. At the end of the sleep study, the PSG recording is divided into 30 s chunks or “sleep epochs”. The five stages (N1, N2, N3, REM, and W) are then assigned to each of the epochs by one or more specialists after qualitative and quantitative inspection of the PSG signals in the frequency and time domains. In most cases, sleep scoring is done in accordance with the AASM [3] or R&K (Rechtschaffen and Kales) [6] standards. In the R&K standards, Stage N3, also often denoted by Slow Wave Sleep (SWS), is divided into two independent stages, N3 and N4.

PSG is the gold standard for assessing sleep problems and quality, but it still calls for unpleasant diagnostic tools with several sensors, experienced observers, and restricted accessibility. As a result, numerous studies have attempted to develop automated algorithms for sleep stage scoring based on multiple inputs such as EOG, EMG, and EEG [7,8,9], or single-channel EEG [10,11,12]. These techniques begin by separating frequency-domain, time-domain, and time–frequency-domain features out of each recording epoch [13,14,15]. After that, the features are passed into conventional machine learning classifiers to determine the epoch’s sleep state [16,17,18].

Recently, deep neural networks have shown excellent performance in several domains including image recognition, natural language processing, and reinforcement learning [19,20,21]. The availability of large amounts of data and high computational capabilities are key enablers for the success of these methods. Thus, end-to-end deep learning frameworks have been developed in order to learn underlying complex patterns in data sets. Recent studies showed increased interest in the use of deep learning in sleep medicine applications [22,23,24,25]. Some studies used deep learning in the form of convolutional neural networks (CNN), which are used mainly with images [22,23]. Other studies used deep learning in the form of recurrent neural networks (RNN) to learn time dependencies in sequential data [24,25]. Similarly, deep learning algorithms have been used for automated detection of sleep stages [10,26,27,28,29].

Despite the remarkable achievements in using deep learning models in sleep stage classification compared to classical learning methods, they still suffer from significant limitations. First, complex deep learning networks with a large number of hidden layers have often been avoided despite the availability of a large number of sleep EEG recordings [30]. Of course, as the number, size, and complexity of the hidden layers increase, the number of training parameters and the training time will increase considerably. In fact, training very complex deep networks from scratch usually requires significant time and considerable computational resources. This also applies to parameter optimization and hyper-parameter tuning (architecture, learning rates, dropout rates, etc.), which also greatly affects performance and requires a considerable amounts of time and experiments for verification. This can also be added to the classical class imbalance problem present in sleep scoring data sets, which poses additional limitations on training deep learning scenarios from scratch. These challenges can significantly limit the use of deep learning methods towards reaching an expert-level performance for sleep stage classification.

The main contributions of our study are as follows:

- We develop a deep transfer learning (DTL) framework for automated scoring of sleep stages using a single EEG channel only. This eliminates the need to establish and train a deep neural network from scratch by using a pre-trained deep neural network architecture that has been previously trained from sufficient labeled data in a different context.
- We investigate the proposed DTL framework with different detection and training scenarios. These include CNN and CNN-RNN architectures, as well as considering different choices for generating imaging data inputs from the corresponding EEG sleep epochs.
- Finally, we evaluate the DTL model on three different EEG single channel inputs. We perform a thorough comparison between the performance achieved over each of the signals considered using different detection and training scenarios.

The structure of this research paper is as follows. Section 2 discusses previous studies in automatic sleep stage scoring. The data sets used in this research are described in Section 3. The proposed deep transfer learning (DTL) framework and the study’s evaluation measures are covered in Section 4. Results for the suggested framework are discussed in Section 5, and they are further examined and analyzed in Section 6. Finally, Section 7 outlines this paper’s conclusions.

## 2. Related Work

Many previous studies considered automated detection of sleep stages using single-channel EEG signals. Nevertheless, the majority of these studies rely on the use of feature engineering methods and classical machine learning algorithms for classifying the different sleep stages based on hand-crafted EEG features. For example, [16,31,32] used support vector machines (SVM), [17,33] used random forests, and [18,29] used recurrent neural networks. Other studies evaluated more than one classifier [11] or used more complex methods such as Dendogram SVM (DSVM) [7] and bootstrap aggregating [33,34,35]. Although these methods have reported a reasonable performance, they carry several serious limitations including the need for sufficient domain expertise and a prior knowledge of sleep analysis as well as a significant time and effort to carefully develop expert-defined features. More importantly, the hand-crafted features are highly dependent on the characteristics of the available data sets. Consequently, this limited the ability of classical machine learning methods to generalize to large patient populations due to the heterogeneity among subjects and recording devices.

This study eliminates the need for complex EEG pre-processing algorithms or the need for human-engineered features, which are required to perform scoring with classical classification methods. The proposed approach advances the state-of-the-art by developing an end-to-end deep learning framework for automatic feature extraction and detection for sleep stages using a single EEG channel. In this regard, few similar studies exist on the use of deep learning instead of conventional algorithms for automated sleep stage scoring. In particular, refs. [2,5,10,27,36,37] presented different end-to-end deep learning approaches for automatic sleep stage scoring using single EEG channel inputs. Although [29] considered a deep recurrent network structure, they used expert-defined features and so cannot be considered an end-to-end approach. There are other deep learning methods that considered multiple PSG signals together with EEG as inputs to their models [26,28,38]. For example, EEG and EOG were considered in [28] and together with EMG in [38]. Additionally, ref. [26] considered six EEG channels simultaneously as inputs to their model. Yet, a major limitation in multi-channel deep learning-based studies is that they can only be implemented in dedicated sleep centers, compared to single EEG algorithms that can also be applied in home or low-resource settings.

The present study provides two aspects of improvement compared to previous end-to-end deep learning approaches for automatic sleep scoring with single EEG channels. First, the proposed system considers a one-to-one classification scheme compared to other studies that used many-to-one [2,10,27,36] or (less frequently) many-to-many [5] classification schemes. The proposed classification system receives a single PSG epoch as an input at a time and produces a single corresponding output label for the sleep stage. This is a much more efficient classification scheme than the many-to-one scheme that augments the classification of the target epoch by combining it with surrounding epochs or the many-to-many scheme that maps an input sequence of multiple epochs to the sequence of their corresponding target labels. Although using many-to-one and many-to-many classification models potentially improved the overall performance by taking into account the existing temporal dependencies between PSG epochs, these approaches suffer from modeling ambiguity and high computational overhead. More importantly, using these schemes poses a major limitation for the development of online and realtime sleep monitoring applications.

The second aspect of improvement in the proposed study compared to previous similar studies is the extensive use of deep transfer learning in building the proposed framework, eliminating the computational overhead required to set up and adequately train a deep learning scoring system from scratch. Among previous similar deep learning studies [2,5,10,27,36,37], the study of [2] was the only one that used transfer learning of a pre-trained CNN applied to a small single data set and evaluated over a single EEG source.

In this study, a comprehensive end-to-end deep transfer learning framework was developed for automated scoring of sleep stages using a single EEG channel. The proposed framework employs an efficient one-to-one classification scheme and extensively uses transfer learning in several training and detection scenarios that are comprehensively evaluated over three EEG signals from two data sets that have different properties and are recorded with different hardware.

## 3. Data Sets

In this study, we leverage two PSG data sets from two major health centers in Germany and USA:

1. DS-1: the first data set, composed of the PSG data for 20 patients that were collected at the Interdisciplinary Center of Sleep Medicine in Charité–Universitätsmedizin Berlin in Berlin, Germany. The polysmongraphy device used to collect these data is manufactured by SOMNO MEDICS (Randersacker, Germany), model: SOMNOscreen PLUS. The data set was approved by the Institutional Ethics Committee at Charité.
2. DS-2: the second data set, which includes the PSG for 61 patients recorded at the Sleep Disorders Center in the University of Michigan in Ann Arbor, Michigan, in the USA. The polysomnography system used for collecting this data set is manufactured by COMPUMEDICS Limited (Victoria, Australia), model: GRAEL PLUS. The Institutional Review Board (IRB) at the University of Michigan approved this study (IRB#HUM00069035).

Each of the polysmnography devices used to collect sleep data from the two health centers included electrodes for electroencephalography (EEG), electrooculography (EOG), electrocardiography (ECG), and submental and tibial electromyography (EMG). The EEG electrodes for DS-1 are reusable gold cup electrodes made by GVB-geliMED (Bad Segeberg, Germany). They either have no brand or GRASS brand. The EEG electrodes for DS-2 are NATUS GRASS brand gold-plated electrodes made by BESDATA (Shenzhen, China)

EEG electrodes were placed using the 10–20 system [3]. The recommended primary EEG channels for sleep scoring according to the AASM, namely F4-M1, C4-M1, and O2-M1, were considered in this study. Sleep scoring was carried out by expert clinicians according to recommendations of the AASM [3]. For each 30 s epoch in the PSG data file of each patient, one of five possible stages is scored by an expert clinician. The scored sleep stage can be either W, N1, N2, N3, or REM. A complete sleep cycle starts with stage W and ends in stage REM.

The PSG studies are standard sleep studies that were conducted on adult subjects in both sleep centers to determine the presence of sleep disorders and possible treatment options. The two data sets were used in previous research to analyze and detect sleep apneic events using respiratory PSG signals [25,39].

The EEG data in DS-1 were sampled at 128 Hz, whereas the EEG data in DS-2 were sampled at 256 Hz. Thus, the EEG data in DS-2 were re-sampled at 256 Hz so that all data have the same sample rate, which translates to a vector with a length of 7680 samples for each EEG sleep epoch. Furthermore, the EEG data was filtered using a zero-phase digital bandpass filter in the range of 0.3–35 Hz, which includes the frequency bands of interest for scoring sleep stages. Table 1 summarizes the detailed distribution for different sleep stages in the data sets. In total, 72,496 sleep epochs were found across the total 81 subjects in this study. The data set was divided randomly such that 90% of the sleep epochs were used for training the different deep learning scenarios while the other 10% of the epochs were used for evaluating the performance of these models in sleep stage classification.

## 4. Materials and Methods

### 4.1. Time–Frequency (TF) Imaging

We used time–frequency imaging to convert raw EEG data into images. Time–frequency (TF) imaging is a technique that allows looking at both the time and frequency domains of a signal simultaneously, using various time–frequency representations. This tool is considered in this research for analyzing EEG signals since different time–frequency patterns are specific to different sleep stages. Time–frequency imaging can be obtained using different methods. In this study, we selected two popular techniques, Fourier-Based Synchrosqueezing Transform (FSST) and Continuous Wavelet Transform (CWT), in order to generate TF images for different sleep EEG epochs.

#### 4.1.1. Fourier-Based Synchrosqueezing Transform (FSST)

The first technique is the FSST, which converts the EEG signal into a time–frequency domain signal. In the time–frequency plane, this approach effectively represents multi-component signals in a condensed manner [40]. It can identify specific time-localized signal components and examine their frequency and variational behavior. By reassigning the coefficients in scale or frequency, this transform, which is a member of the family of time–frequency reassignment techniques (RM), operates on the time–frequency domain of the Short Time Fourier Transform (STFT). By moving components to a neighboring ridge, commonly referred to as the energy distribution’s center of gravity, this RM technique has been used to sharpen spectrograms. It is simpler to discern between EEG epochs at different stages of sleep thanks to this relocation process, which produces sparse and sharpened time–frequency representations. Other time–frequency representations can also use the reassignment, provided that the coefficients are reassigned for both the time and frequency components [41]. To maintain causality, only the frequency component of the FSST’s coefficients is redistributed.

Many physiological signals can be expressed as a superposition of amplitude-modulated and frequency-modulated modes. For time–frequency analysis, the FSST decomposes an EEG sleep epoch y(t) as a multi-component signal consisting of *K* oscillatory components defined by [40,41]:

(1) $$y\left(t\right)=\sum_{k=1}^{K}y_{k}\left(t\right)=\sum_{k=1}^{K}A_{k}\left(t\right)e^{j2\pi \phi_{k}\left(t\right)}$$

where $A_{k}\left(t\right)$ is the instantaneous amplitude and $\phi_{k}^{\prime }\left(t\right)$ (derivative of the phase) is the instantaneous frequency of component *k*. For a weak frequency modulation between components, there exists a small value ϵ≪1, $∥A_{k}^{\prime }\left(t\right)∥\ll \epsilon \phi_{k}^{\prime }\left(t\right)$ and $∥\phi_{k}^{\prime }\left(t\right)∥\ll \epsilon \phi_{k}^{\prime \prime }\left(t\right)$. This requires amplitude to be differentiable and phase to be twice differentiable. The adjacent components are well-separated in frequency with a distance *d*, $\phi_{k}^{\prime }\left(t\right)-\phi_{k-1}^{\prime }\left(t\right)>d$. For a Gaussian window *g* of size $\gamma_{g}$, the frequency bandwidth of *g* is $\Delta =\sqrt{2}\frac{log(2)}{\gamma_{g}}$. Accordingly, the minimum distance between adjacent components is d=2Δ.

The FSST $T_{f}\left(w,t\right)$ is based on the modified coefficients of Short-Time Fourier Transform (STFT) $V_{f}\left(\eta ,t\right)$ from (η,t) to $\left({\hat{\omega }}_{f}\left(\eta ,t\right),t\right)$ described by the synchrosqueezing operator:

(2) $$T_{f}\left(w,t\right)=\frac{1}{g(0)}\int V_{f}\left(\eta ,t\right)\delta \left(\omega -{\hat{\omega }}_{f}\left(\eta ,t\right)\right)d\eta$$

where g(0) is the value of a sliding window g(t) at time 0, δ is the Dirac delta function, and ${\hat{\omega }}_{f}\left(\eta ,t\right)$ is the instantaneous frequency defined by:

(3) ω^f(η,t)=Re1j2π∂tVf(η,t)Vf(η,t)

The instantaneous frequency can be approximated by ${\hat{\omega }}_{f}\left(\eta ,t\right)$, when $V_{f}\left(\eta ,t\right)>0$. Moreover, from the FSST, we can obtain the complex-valued bivariate image $T_{f}\left(\omega ,t\right)$ for each EEG sleep epoch.

#### 4.1.2. Continuous Wavelet Transform (CWT)

CWT is a useful method for representing time series at various resolutions. Using CWT, a time series can be transformed mathematically into a different feature space in order to be employed in feature extraction in the time–frequency domain [41]. By performing a mathematical inner product operation on the signal and a collection of wavelets, the wavelet transform is produced. This group of wavelets is a wavelet family that was created by scaling and translating the mother wavelet ψ(t), which can be represented as:

(4) $$\psi_{s,\tau }\left(t\right)=\frac{1}{\sqrt{s}}\psi \left(\frac{t-\tau }{s}\right)$$

where τ is a translation parameter and *s* is a scale parameter inversely related to frequency.

A CWT of an EEG sleep epoch y(t) can be obtained by a convolution operation with a complex conjugate, mathematically defined as follows:

(5) $$W\left(s,\tau \right)=\langle y\left(t\right),\psi_{s,\tau }\left(t\right)\rangle =\frac{1}{\sqrt{s}}\int \psi^{*}\left(\frac{t-\tau }{s}\right)dt$$

where $\psi^{*}\left(.\right)$ denotes the complex conjugate of ψ(.). This operation decomposes the EEG sleep epoch y(t) into a series of wavelet coefficients where the wavelet family is the basis function. Observing the above equations, there are two types of parameters in family wavelets: *s* and τ. Through the convolution operation, the sleep epoch segment y(t) is transformed by the family wavelets and projected to the two-dimensional (2D) time and scale dimensions [41]. Accordingly, successive one-dimensional EEG sleep segments are converted into TF images.

### 4.2. Convolutional Neural Networks (CNNs)

A CNN is a feedforward neural network with a deep structure and is one of the popular representative algorithms of deep learning. They are widely used when dealing with image tasks. Using raw input 2D images in CNN architectures is the standard in applications of deep learning in computer vision and signal processing [42,43,44,45,46]. Thus, in this study we used TF images generated from raw EEG sleep epochs as inputs to the CNN.

Deep CNNs have the ability to automatically and efficiently learn hierarchical features from input images, such that the higher-level layers’ features are more abstract than the lower layers’. Convolution layers (filtering), pooling layers (subsampling) with a type of nonlinearity applied before or after pooling, and lastly fully-connected layer(s) are the types of layers that typically make up a CNN. Convolution blocks, which are created by combining convolution and pooling layers, are often stacked to create a deep architecture. In classification tasks, a softmax (multinomial logistic regression) layer is commonly added to CNNs with a size that is equal to the number of target classes. CNNs are trained using iterative optimization with the backpropagation algorithm. The most common optimization method in the literature is stochastic gradient descent (SGD). More details about the definition and computational processes in CNNs are introduced in [47].

### 4.3. Recurrent Neural Networks (RNNs)

Recurrent Neural Networks (RNNs) are a distinct class of neural networks that excel at handling time-series data and are well-suited for sequential information, as opposed to ordinary feedforward networks that take each input value of a signal into account independently [24].

**Figure 1:**
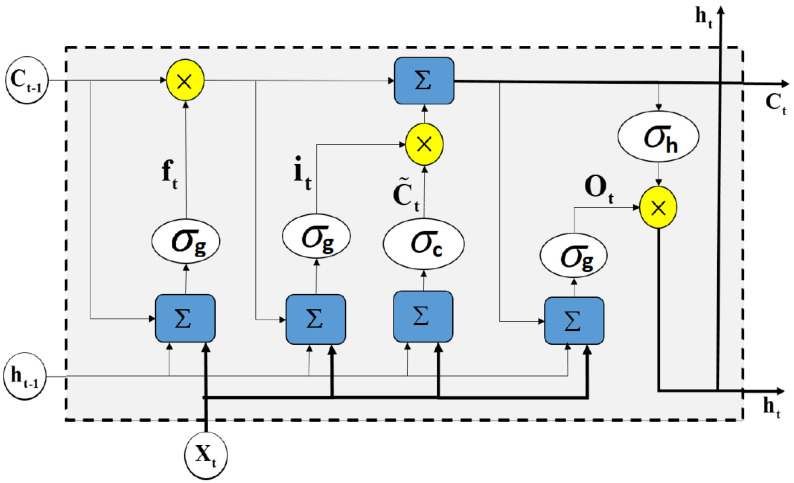
A typical architecture of an LSTM cell. An LSTM block typically has a memory cell, input gate ($i_{t}$), output gate ($O_{t}$), and a forget gate ($f_{t}$) in addition to the hidden state ($h_{t}$) in traditional RNNs [25].

However, due to the vanishing and exploding gradient problem [48], traditional RNNs are unable to detect long-range dependencies. The aforementioned issue is addressed by the Long Short-Term Memory (LSTM) network, which is an expanded version of RNN equipped with a gating mechanism in order to regulate the flow of information. It also has the ability to extract deeper contextual data from time series. Because we conducted a retrospective analysis of the PSG recordings in this investigation, we were able to apply a Bidirectional LSTM (BiLSTM) variation. Causal and anticausal counterpart LSTM layers make up each BiLSTM layer. Figure 1 depicts a single causal LSTM unit that processes the time series forward in time. This unit can be formally described as follows:

(6) $$\begin{array}{ccc} f_{t} & = & \sigma_{g}\left(W_{f}x_{t}+U_{f}h_{t-1}+b_{f}\right) \end{array}$$

(7) $$\begin{array}{ccc} i_{t} & = & \sigma_{g}\left(W_{i}x_{t}+U_{i}h_{t-1}+b_{i}\right) \end{array}$$

(8) $$\begin{array}{ccc} C_{t} & = & f_{t}⊙C_{t-1}+i_{t}⊙\sigma_{c}\left(W_{c}x_{t}+U_{c}h_{t-1}+b_{c}\right) \end{array}$$

(9) $$\begin{array}{ccc} O_{t} & = & \sigma_{g}\left(W_{o}x_{t}+U_{o}h_{t-1}+b_{o}\right) \end{array}$$

(10) $$\begin{array}{ccc} h_{t} & = & O_{t}⊙\sigma_{h}\left(C_{t}\right) \end{array}$$

where Equations (6)–(10) represent respective mathematical models for the forget gate, input gate, cell state update, output gate, and output function of an LSTM unit with an input vector $x_{t}$, respectively, while $C_{t-1}$ is the state and $h_{t-1}$ is the output of the preceding LSTM unit. For each gate *∈{i,f,g,c}, $b_{*}$ is the bias term, $W_{*}$ is the input weight matrix, and $U_{*}$ is the recurrent weight matrix. These are the training parameters that are updated during the network learning process. The operator ⊙ in Equations (6)–(10) is the Hadamard product. $\sigma_{c}$ and $\sigma_{h}$ are tangent hyperbolic activation functions, while $\sigma_{g}$ is the sigmoid activation function.

To process the time series backwards in time, the BiLSTM has an anticausal (reverse) LSTM in addition to the forward LSTM. The anticausal LSTM is very similar to the causal LSTM but with a reverse time order. Thus, Equations (6)–(10) can be used after replacing $b_{*}$, $W_{*}$, and $U_{*}$ with $W_{*}^{\prime }$, $U_{*}^{\prime }$, and $b_{*}^{\prime }$, respectively, as well as replacing $h_{t-1}$ and $C_{t-1}$ with $h_{t+1}^{\prime }$ and $C_{t+1}^{\prime }$, respectively. In order to capture bidirectional long-term relationships between time steps of the time series, the outputs of the forward and reverse LSTMs are concatenated in the final step.

### 4.4. Transfer Learning and Fine Tuning Strategy

In learning theory, transfer Learning ($T_{L}$) is an approach that targets applying the knowledge learned from one task to another new but relevant task in order to enhance the learning performance of the new task. The domain of the original task is called the source domain ($D_{s}$), while the domain of the new task is called the target domain ($D_{t}$) [49]. By initializing the target model using parameters that are transferred from a pre-trained model, $T_{L}$ is able to aid in the training of a target model. Furthermore, $T_{L}$ is a very effective method for the quick building and evaluation of deep learning models when training a deep architecture from scratch is complicated.

Complex deep neural networks frequently have a lot of weights, which are iteratively updated based on labeled data and a loss function after being randomly initialized prior to training. This iterative procedure is extremely time-consuming with all labeled data. Additionally, in cases with limited training data, deep architectures are prone to overfit to the training data. Instead of creating and training a CNN from scratch, $T_{L}$ offers a simple solution that uses a pre-trained deep CNN that was already trained by another data set.

Several studies have shown effectiveness of transfer learning with pre-trained models in medical imaging applications [50] as well as fault detection applications of mechanical systems [51,52]. In this study, we leveraged GoogLeNet as a pre-trained CNN to investigate knowledge transfer from natural images to time–frequency (TF) images of sleep epochs in EEG data.

### 4.5. A Framework for Automatic Sleep Staging Using Deep Transfer Learning

The proposed framework is based on deep transfer learning where time–frequency images of single-channel EEG sleep epochs are used as the input. $T_{L}$ based on pre-trained model helps improve deep model performance. This study proposes an end-to-end deep learning framework that is automatically able to learn features and recognize sleep stages using single-channel EEG signals. Our framework is composed of three stages: Time–frequency imaging, pre-trained model building with fine-tuning, and finally model application.

Three EEG signal channels acquired by PSG were evaluated in this study, including C4-M1, F4-M1, and O2-M1, which are the primary signals recommended by AASM for sleep scoring. Data from each of these signals were segmented at 30 s sleep epochs. EEG epochs were then transformed from the time domain to the time–frequency domain, forming a set of time–frequency images that were utilized as the input to the following pre-trained model. In this study, two methods were evaluated for time frequency imaging: FSST and CWT.

The pre-trained CNN model used in this paper is GoogLeNet, which is a deep convolutional network created by Google. It is 22 layers deep and was originally trained on the ImageNet data set [53] in order to classify images into 1000 object categories, such as keyboard, mouse, pencil, and many animals. This network has originally achieved accurate classification performance on the ImageNet data set and was chosen for this study since it provides a good tradeoff between classification accuracy and computational complexity [54]. The pre-trained model was trained on the ImageNet data set, but the target data set was the time–frequency images of EEG sleep epochs.

**Figure 2:**
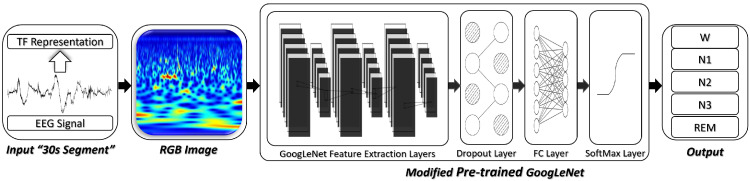
Deep CNN transfer learning approach for automatic scoring of sleep stages.

**Figure 3:**
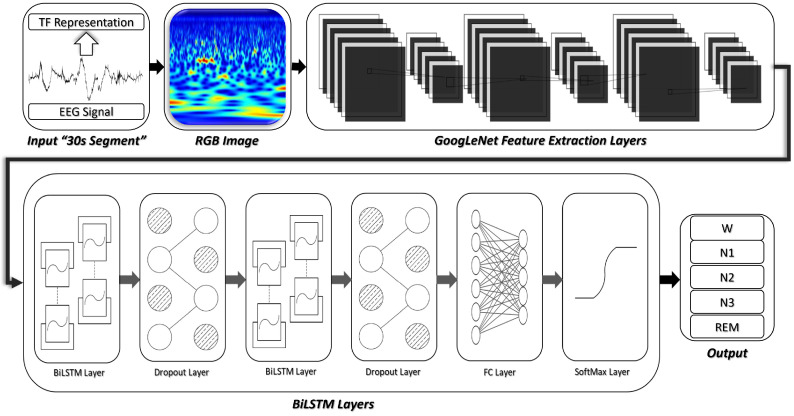
Deep CNN-RNN transfer learning based approach for automatic scoring of sleep stages.

As shown in Figure 2 and Figure 3, we considered two deep transfer architectures for automatic scoring of sleep stages. The first one is a CNN-based transfer learning approach. In this case, the feature-extraction layers of the pre-trained model were transferred and a dropout layer was added before the fully connected layer. Finally, the final layer of the pre-trained CNN model was replaced with a softmax output layer whose size is dictated by the number of target sleep stages (five classes). Weights of the new output layer were initialized randomly. During the training process, weights of the trainable layers were updated to minimize errors between predicted labels and the true ones. After enough epochs, the designed model was fine-tuned and the deep CNN-based architecture together with all the of parameters were saved.

The second architecture, illustrated in Figure 3, is a CNN-RNN-based transfer learning approach. First, the feature-extraction layers of the pre-trained CNN model were transferred. Then, a recurrent neural network was added to form a CNN-RNN deep architecture. The recurrent network part of the CNN-RNN is composed of two BiLSTM layers and each of them is followed by a dropout layer to avoid overfitting. Finally, a softmax output layer with five nodes was added to enable classification between the five possible sleep stages. In order to convert successive time–frequency images to sequences of feature vectors, restore the sequence structure, and reshape the output to vector sequences, we used a sequence unfolding layer and a flatten layer prior to the RNN part of the network. During the training process, weights of the trainable layers were updated to minimize errors between predicted scores and the corresponding clinical annotations. After enough epochs, the designed model was fine-tuned and the deep CNN-RNN-based architecture together with all corresponding parameters were saved.

This process was performed on EEG training segments (90% of data), to build and fine-tune each of the proposed deep transfer approaches through evaluating each of the time–frequency imaging approaches on each of the three EEG signal channels investigated in this study. The testing data set (10% held-out EEG data) was then used to validate the ability of the proposed framework to perform automated scoring of sleep stages and to compare performance considering different signal inputs, time–frequency imaging methods, and modeling scenarios. To avoid excessive computational loads and processing times, we did not initially over-sample the training data (due to the class imbalance between sleep stages) so that all different options can be evaluated in a reasonable time. Subsequently, we applied over-sampling to train the detection scenario that showed best results. Finally, we used 20-fold cross-validation over the input signal that showed the best scoring performance among the three input signals considered in this study in order to report a comprehensive evaluation for the proposed framework.

### 4.6. Evaluation Metrics

Recognizing the classical high imbalance problem in sleep scoring data, the proposed framework was evaluated for both per-class performance and for the overall performance to ensure a comprehensive evaluation of the proposed methods.

Per-class metrics that were computed in this study include sensitivity, precision, F1-score, specificity, and accuracy. The per-class metrics were computed by considering a single class as a positive class and all other classes combined as a negative class. Thus, for each of the five classes of interest, a one-versus-all classification problem was considered to compute true positives (TP), true negatives (TN), false positives (FP), and false negatives (FN). Accordingly, per-class metrics can be computed from the following equations:

(11) $$\begin{array}{ccc} Sn_{c} & = & \frac{TP}{TP+FN}\times 100\% \end{array}$$

(12) $$\begin{array}{ccc} Pr_{c} & = & \frac{TP}{TP+FP}\times 100\% \end{array}$$

(13) $$\begin{array}{ccc} F1_{c} & = & 2\frac{Sn.Pr}{Sn+Pr}\times 100\% \end{array}$$

(14) $$\begin{array}{ccc} Sp_{c} & = & \frac{TN}{FP+TN}\times 100\% \end{array}$$

(15) $$\begin{array}{ccc} ACC_{c} & = & \frac{TP+TN}{TP+FP+TN+FN}\times 100\% \end{array}$$

where $Sn_{c}$, $Pr_{c}$, $F1_{c}$, $Sp_{c}$, and $ACC_{c}$ are per-class sensitivity, precision, F1-score, specificity, and accuracy of class c∈{1,2,...,C}, respectively, and C=5 is the number of sleep stages.

For the overall classification metrics, we considered overall accuracy (ACC), macro-average F1 (MF1), overall sensitivity (Sn), and overall specificity (Sp). These metrics can be mathematically expressed as follows:

(16) $$\begin{array}{ccc} ACC & = & \frac{\sum_{c=1}^{C}TP_{c}}{N}\times 100\% \end{array}$$

(17) $$\begin{array}{ccc} MF1 & = & \frac{\sum_{c=1}^{C}F1_{c}}{C} \end{array}$$

(18) $$\begin{array}{ccc} Sn & = & \frac{\sum_{c=1}^{C}Sn_{c}}{C} \end{array}$$

(19) $$\begin{array}{ccc} Sp & = & \frac{\sum_{c=1}^{C}Sp_{c}}{C} \end{array}$$

where $TP_{c}$ is the true positives of class c∈{1,2,...,C} and *N* is the total number of test epochs. To compare results across different detection scenarios and different EEG signal channels, per-class sensitivity ($Sn_{c}$) is reported as well as the per-class F1-score ($F1_{c}$), which provides a comprehensive snapshot of the per-class performance by considering the sensitivity/precision tradeoff. Additionally, the overall performance across different detection scenarios and different EEG signal channels was also compared using the accuracy (ACC), macro-average F1 (MF1), overall sensitivity (Sn), and overall specificity (Sp). Finally, the detailed performances for the best-performing scenarios were thoroughly analyzed using all per-class metrics: sensitivity ($Sn_{c}$), specificity ($Sp_{c}$), precision ($Pr_{c}$), F1-score ($F1_{c}$), and per-class accuracy ($ACC_{c}$).

## 5. Results

### 5.1. TF Imaging Data

**Figure 4:**
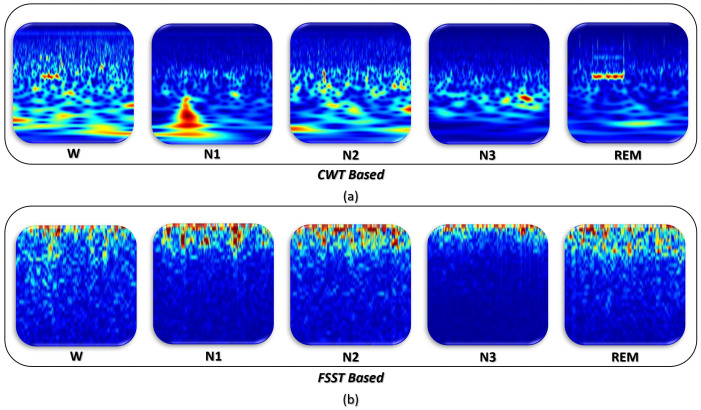
TF-images from 30 s EEG epochs in five different sleep stages (**a**) obtained with CWT and (**b**) obtained with FSST.

First, the EEG signals were pre-processed using two types of TF image representations: The FSST power spectrum and the CWT scalogram. Each of these representations were obtained for consecutive 30 s EEG Epochs. The TF images were then re-scaled to the size of 224×224×3 as required by the GoogLeNet CNN model. Finally, the processed images were divided into two parts: the training data set and the testing data set. The training data set was used to train and fine-tune network weights of the pre-trained model, whereas the testing data set was only used to verify the performance of the deep model and was not used during the training process. The processed TF images obtained with the CWT and FSST methods for each of the sleep stages are shown in Figure 4a,b, respectively.

### 5.2. Performance of Deep CNN Transfer Learning Networks

Table 2 compares the overall performance of the different CNN-based transfer learning networks in automatic scoring for sleep stages. The CNN-based transfer learning architecture was evaluated on the three EEG channels considered in this study, C4-M1, F4-M1, and O2-M1 and using the two TF representations (CWT, FSST). Each model was evaluated for the overall accuracy (ACC), macro F1-score MF1, overall sensitivity (Sn), and specificity (Sp), as well as per-class sensitivity ($Sn_{c}$) and per-class F1-scores ($F1_{c}$).

The results in Table 2 show that the test CNN performance results obtained with CWT-TF image representation are significantly higher for the C4-M1 and F4-M1 signals compared to the FSST-TF method applied to these signals. The CNN transfer learning model built using EEG data from the O2-M1 channel using both TF representation methods showed lower ability to correctly identify sleep stages compared to the performance obtained with C4-M1 and F4-M1.

The highest classification results for the CNN transfer learning approach were achieved using CWT-TF image representations obtained from the C4-M1 EEG channel. The detailed per-class test performance for this best-performing scenario is shown in Table 3. Results are shown for three trials with their mean and standard deviation. The CNN transfer learning model achieved high per-class specificity and accuracy in all classes, a much higher sensitivity in the W and N2 classes compared to other classes, and a generally high precision, except for class N1, which also achieved the lowest per-class sensitivity.

### 5.3. Performance of Deep CNN-RNN Transfer Learning Networks

Table 4 summarizes the performance of the CNN-RNN-based transfer learning approach in automatic scoring for sleep stages, comparing the three EEG sources along with the two TF representation methods implemented in this study. Inspecting this table shows that the CNN-RNN transfer learning models with CWT-TF image representation achieved higher overall performance in sleep scoring compared to those obtained with FSST-TF image representations. Similar to the CNN transfer learning modeling framework, C4-M1 and F4-M1 EEG channels showed an overall improved performance in detecting sleep stages with the CNN-RNN transfer learning approach compared to the detections obtained from O2-M1.

Furthermore, Table 2 and Table 4 can be used for comparing test results obtained with the CNN and CNN-RNN transfer learning approaches. It can be clearly noticed that the CNN-RNN transfer learning scenario provided an overall improved detection performance compared to the CNN transfer learning scenario as illustrated by all of the listed performance metrics. Combining the CNN and RNN networks provides the ability to extract features present in the EEG spectrograms while preserving the temporal relationship present in the EEG data.

The highest classification results for the CNN-RNN transfer learning were achieved with CWT-TF image representation obtained from the C4-M1 EEG channel. The detailed per-class test performance for this best-performing scenario is shown in Table 5. To ensure the robustness of the proposed approach, the results are shown for three trials with their mean and standard deviation. The CNN-RNN transfer learning model achieved significantly higher per-class-sensitivity and precision compared to the CNN transfer learning model for data from the same EEG channel and using the same Time–Frequency (TF) imaging approach. Despite the improvement obtained in per-class sensitivity with the CNN-RNN approach, the lowest per-class sensitivity was still obtained with class N1.

### 5.4. Performance of Deep CNN-RNN Transfer Learning Networks with Oversampled Training Data

Our results indicate an improved performance in automated sleep scoring with the CNN-RNN transfer learning approach over the CNN transfer learning approach. Moreover, time–frequency images as inputs to the deep transfer learning framework showed a better performance with the continuous wavelet transform approach compared to the Fourier-based synchrosqueezing transform. Accordingly, the best detection approach is the CNN-RNN transfer learning modeling framework using the continuous wavelet transform approach for TF-image representation (CWT-TF).

Next, we considered the technique of oversampling to overcome the issue of class imbalance in the distribution of the five different sleep stages in our data set. Thus, we retrained the best detection scenario for the proposed CNN-RNN approach using oversampled CWT-TF training images while still evaluating the model on the same (originally sampled) testing data in order to demonstrate the effect of training with oversampled images as compared to the performance obtained with training with original samples in Table 4. This was repeated over the three EEG channels considered in this study and the results are summarized in Table 6. As expected, training the CNN-RNN modeling framework with oversampled CWT-TF images improved the overall performance over all of the listed EEG channels. It also provided more consistent classification performance across the five different sleep stages. In particular, the oversampling significantly increased the performance of N1, N3, and REM stages. Similar to what was observed in Table 4, Table 6 still indicates an improved scoring performance over the C4-M1 and F4-M1 channel inputs compared to the performance over O2-M1. Similarly, the best-performing scenario in Table 6 is the CNN-RNN transfer learning model that was trained with oversampled CWT-TF training images from the C4-M1 EEG channel.

Finally, in order to ensure the generalizability of the proposed framework over testing data, the best-performing scenario was comprehensively evaluated using 20-fold cross-validation. To avoid excessive computational load, this approach was applied only on the best-performing scenario in Table 6. In this approach, EEG data epochs from C4-M1 were randomly divided into 20 folds where 19 out of the 20 folds were used for building the model that was then evaluated on the remaining fold. The process was repeated twenty times, in which each time the model was built with oversampled data from the training folds while being evaluated on original samples for each of the 20 folds. Table 7 reports the 20-fold cross-validation per-class performance of the proposed CNN-RNN modeling framework applied to oversampled CWT-TF training images obtained from C4-M1. The detailed per-class performance results in Table 7 show an excellent average per-class detection performance over the 20 test folds along with a small standard deviation across these folds, indicating an excellent potential for the proposed model to generalize over unseen EEG data.

## 6. Discussion

This study presents a comprehensive Deep Transfer Learning (DTL) framework for automated scoring of sleep stages using a single EEG channel. Two deep learning architectures were investigated. The first one considers a pre-trained CNN only, whereas the second one considers a CNN-RNN architecture with a BiLSTM network that follows the pre-trained CNN. Experiments showed an improved performance of the CNN-RNN detection scenario in detecting sleep stages compared to the CNN scenario. This improvement is achieved because the BiLSTM network is able to consider temporal dependencies and extract temporal features in EEG data. The cyclic behavior for the occurrence of sleep stages allows the BiLSTM network to enhance the overall performance in detecting sleep stages.

In order to generate imaging data inputs needed for the proposed DTL framework, two popular time–frequency (TF) imaging approaches were applied to the EEG sleep epochs. The Fourier-Based Synchrosqueezing Transform (FSST) and the Continuous Wavelet transform (CWT) were employed to generate TF representations for successive EEG epochs. Our results show an improved performance in detecting sleep stages using the TF representations obtained by CWT compared to those obtained by the FSST approach. This improvement was consistently noticed regardless of the EEG channel source and across all of the deep learning architectures considered. The results demonstrate that the CWT approach provided better localization and concentrated representation for the different frequency components present in the EEG sleep epochs.

The proposed framework was evaluated on the three primary EEG channels recommended by AASM for scoring sleep epochs in PSGs studies (C4-M1, F4-M1, and O2-M1). Our results show that, without changing the model architecture and the training algorithm, the proposed modeling framework can be applied on different EEG signals. Yet, it was noticed that the detection performance achieved with C4-M1 and F4-M1 was significantly better than the detection performance achieved with O2-M1. Interestingly, the study demonstrated that the proposed framework is able to work with EEG data sets from two different data centers with different recording properties and different EEG sampling rates. These results demonstrate that the presented framework is able to generalize well over different hardware settings and different single-channel EEGs.

Furthermore, the proposed framework leverages Deep Transfer Learning (DTL) as an efficient tool for rapid development and evaluation of the proposed framework, eliminating the computational time and effort and complexities required to set up and sufficiently train and fine-tune a deep learning scoring system from scratch. In particular, this study focused on using the existing feature extraction layers of the pre-trained GoogLeNet CNN model for automatic feature extraction from the time–frequency representations of the EEG epochs. The temporal features were also automatically extracted through the RNN part (BiLSTM network). Future work will focus on modifying or adding more feature extraction layers in the pre-trained CNN model in order to evaluate the effect on the overall performance in discriminating between TF images that belong to different sleep stages.

Table 8 compares the performance of the proposed DTL framework with the state-of-the-art deep learning methods. The proposed DTL system composed of a pre-trained CNN followed by an RNN achieved a comparable performance to the studies in Table 8. However, it should be pointed out here that these studies vary with respect to the method that was used for handling the classical imbalance problem in sleep scoring data sets. For example, subsampling approaches were used to generate randomly class-balanced data [10,36] as well as to trim data from the most represented classes [37], which might affect the performance in real case scenarios.

In our analysis, we used the complete data set with the actual imbalanced class distributions. The DTL system composed of a pre-trained CNN followed by an RNN was trained using original EEG samples and also using oversampled data from the same EEG channel, and both scenarios were evaluated on hold out (un-augmented) EEG data. As displayed in Table 8, using oversampled EEG data for training the DTL CNN-RNN framework significantly improved the detection performance for the less-represented stages N1, N3, and REM in our data set. Yet, it also had a (smaller) negative effect on the detection performance of the majority classes W and N2. In practice, stage N1 is known as the most challenging stage to detect since it is a transition stage between wakefulness and sleep. This stage typically lasts only 2–5% of the total duration of a standard sleep cycle and can be misinterpreted as Wake, N2, or REM (even by an expert) [37,55]. This can be further verified by observing the low detection performance achieved with this stage in previous studies listed in Table 8. Interestingly, the proposed approach shows a significant improvement in the ability to detect stage N1. Future research is necessary to develop novel methods to address the class imbalance in order to further improve the overall performance of the proposed framework.

Even though our results are encouraging, there are some limitations to our study. We only considered the analytic Morse wavelet as the mother wavelet in order to generate CWT-TF representations. This allowed maintaining consistency across all of the CWT experiments carried out in this study. We also considered one pre-trained CNN for transfer learning in our experiments. Future work may consider performing a comprehensive analysis over the proposed CNN-BiLSTM model with different wavelet families and different pre-trained CNN networks for improving the performance. We also plan to study the effect of designing an ensemble of different pre-trained CNNs to improve the overall performance in sleep scoring.

## 7. Conclusions

We propose an end-to-end deep transfer learning framework for automated scoring of sleep stages based on single-channel EEG signals without the need for any human-engineered features. Two time–frequency imaging approaches were investigated in order to obtain the time–frequency representations of EEG sleep epochs. Additionally, several detection and training scenarios were comprehensively evaluated, including CNN compared to CNN-RNN architectures. The best detection results where obtained using a deep architecture composed of the GoogLeNet CNN followed by a BiLSTM network that operates on time–frequency images generated with the continuous wavelet transform applied to the EEG sleep epochs. Furthermore, the proposed system is set up and trained using transfer learning in order to eliminate the computational overhead and experience required to set up and sufficiently train a deep learning scoring system from scratch.

Our results demonstrate that the proposed modeling framework is able to automatically learn features and score sleep stages in three different single-channel EEGs obtained from two completely different data sets. The proposed CNN-BiLSTM system achieved promising performance while using a one-to-one classification scheme, making it suitable for online and real time monitoring applications.

Future efforts will focus on improving the proposed system in order to be applied to single-channel EEG sources recorded by wearable devices.

## Figures and Tables

**Table 1:** Distribution of sleep stages corresponding to different sleep epochs in the data sets used in the study.

PSG	Center	Patients	Number (Percentage) of Sleep Stage Epochs					Total
			W	N1	N2	N3	REM	
DS-1	Charité	20	4826	5094	4711	1568	1488	17,687
			(27.29%)	(28.80%)	(26.64%)	(8.87%)	(8.41%)	
DS-2	Umich	61	12,983	5818	26,091	3621	6296	54,809
			(23.69%)	(10.62%)	(47.60%)	(6.61%)	(11.49%)	
	Total	81	17,809	10,912	30,802	5189	7784	72,496
			(24.57%)	(15.05%)	(42.49%)	(7.16%)	(10.74%)	

**Table 2:** Overall test performance results for the CNN transfer learning networks over different EEG channels and different time–frequency imaging approaches.

Original Sampling		Overall Metrics				Per-Class Sensitivity (${\mathrm{Sn}}_{c}$)					Per-Class F1 ($F1_{c}$)				
**Sig.**	**TF Rep.**	ACC	MF1	Sn	Sp	**W**	**N1**	**N2**	**N3**	**REM**	**W**	**N1**	**N2**	**N3**	**REM**
C4-M1	FSST	73.9	67.3	66.3	92.6	88.4	35.3	84.1	59.5	64.2	85.6	41.8	80.0	64.2	65.1
F4-M1	FSST	74.8	68.5	67.8	92.8	87.0	33.2	85.3	61.1	72.3	85.8	39.5	80.9	65.8	70.6
O2-M1	FSST	72.2	64.9	63.6	92.0	86.7	34.4	84.0	57.9	54.8	85.7	40.3	78.7	62.8	56.8
**C4-M1**	**CWT**	**75.4**	**70.0**	**68.8**	**93.0**	**85.8**	**37.9**	**85.9**	**65.5**	**69.1**	**83.3**	**44.9**	**81.7**	**70.2**	**69.8**
F4-M1	CWT	75.1	69.6	68.8	92.9	84.5	32.2	86.3	63.0	77.8	82.3	39.3	81.6	68.7	76.3
O2-M1	CWT	71.8	65.1	63.8	91.9	84.2	31.0	84.0	59.3	60.5	81.8	37.6	78.7	65.4	62.0

The bold rows in the tables show the best performing scenarios which are further discussed in the text.

**Table 3:** Detailed per-class best test performance obtained with CWT-TF representation from C4-M1 using CNN transfer learning network. Results are reported as the mean (std) for three trials.

Class	${\mathrm{Sn}}_{c}$	${\mathrm{Sp}}_{c}$	${\mathrm{Pr}}_{c}$	$F1_{c}$	${\mathrm{ACC}}_{c}$
W	85.8 (0.4)	93.4 (0.3)	80.9 (0.7)	83.3 (0.5)	91.6 (0.3)
N1	37.9 (1.6)	94.5 (0.3)	55.1 (0.8)	44.9 (1.1)	86.0 (0.2)
N2	85.9 (0.6)	81.9 (0.5)	77.8 (0.4)	81.7 (0.2)	83.6 (0.2)
N3	65.5 (2.0)	98.4 (0.2)	75.7 (1.9)	70.2 (1.0)	96.0 (0.1)
REM	69.1 (1.4)	96.5 (0.1)	70.6 (0.8)	69.8 (1.0)	93.6 (0.2)

**Table 4:** Overall Test performance results obtained using CNN-RNN transfer learning networks over different EEG channel inputs and different time–frequency imaging approaches.

Original Sampling		Overall Metrics				Per-Class Sensitivity (${\mathrm{Sn}}_{c}$)					Per-Class F1 ($F1_{c}$)				
**Sig.**	**TF Rep.**	ACC	MF1	Sn	Sp	**W**	**N1**	**N2**	**N3**	**REM**	**W**	**N1**	**N2**	**N3**	**REM**
C4-M1	FSST	75.0	69.3	68.5	92.9	88.0	40.4	83.7	63.6	66.6	86.3	45.3	80.7	67.0	66.9
F4-M1	FSST	75.7	70.2	69.7	93.1	87.5	38.2	84.5	62.9	75.3	86.5	43.6	81.4	67.0	72.5
O2-M1	FSST	72.9	66.2	65.0	92.3	86.7	37.3	83.5	60.0	57.7	85.9	42.5	79.1	64.1	59.2
**C4-M1**	**CWT**	**77.3**	**73.0**	**72.0**	**93.5**	**85.3**	**44.3**	**86.2**	**68.9**	**75.4**	**84.4**	**50.3**	**82.6**	**73.2**	**74.5**
F4-M1	CWT	76.4	71.7	70.8	93.3	84.5	38.6	86.1	65.8	78.8	83.4	44.5	82.4	70.9	77.4
O2-M1	CWT	73.3	67.7	66.7	92.4	84.3	34.7	83.8	62.9	67.7	82.6	40.9	79.6	68.4	67.2

The bold rows in the tables show the best performing scenarios which are further discussed in the text.

**Table 5:** Detailed per-class best test performance obtained with CWT-TF representation from C4-M1 using CNN-RNN transfer learning network. Results are reported as the mean (std) for three trials.

Class	${\mathrm{Sn}}_{c}$	${\mathrm{Sp}}_{c}$	${\mathrm{Pr}}_{c}$	$F1_{c}$	${\mathrm{ACC}}_{c}$
W	85.3 (0.3)	94.6 (0.3)	83.6 (0.6)	84.4 (0.2)	92.3 (0.1)
N1	44.3 (1.4)	94.4 (0.1)	58.2 (1.0)	50.3 (1.3)	86.8 (0.3)
N2	86.2 (0.3)	83.4 (0.3)	79.3 (0.3)	82.6 (0.3)	84.6 (0.2)
N3	68.9 (1.0)	98.5 (0.0)	78.1 (0.2)	73.2 (0.6)	96.4 (0.1)
REM	75.4 (0.3)	96.8 (0.1)	73.7 (0.7)	74.5 (0.4)	94.5 (0.1)

**Table 6:** Overall test performance with CNN-RNN transfer learning networks and oversampled CWT-TF training images across three different EEG channel inputs.

Oversampled Data		Overall Metrics				Per-Class Sensitivity (${\mathrm{Sn}}_{c}$)					Per-Class F1 ($F1_{c}$)				
**Sig.**	**TF Rep.**	ACC	MF1	Sn	Sp	**W**	**N1**	**N2**	**N3**	**REM**	**W**	**N1**	**N2**	**N3**	**REM**
**C4-M1**	**CWT**	**76.9**	**75.7**	**77.1**	**93.9**	**79.0**	**69.5**	**77.0**	**80.4**	**79.4**	**83.8**	**57.1**	**81.1**	**76.3**	**80.2**
F4-M1	CWT	76.6	74.9	75.9	93.8	78.7	59.9	79.4	77.8	83.7	82.6	53.3	81.7	75.1	81.9
O2-M1	CWT	74.5	72.5	73.4	93.2	78.4	58.5	77.0	76.8	76.3	82.5	50.9	79.5	73.1	76.4

The bold rows in the tables show the best performing scenarios which are further discussed in the text.

**Table 7:** 20-fold per-class cross validation performance obtained with oversampled CWT-TF training data applied on C4-M1 and using CNN-RNN transfer learning network. Results are reported as the mean (std) across the 20-fold results.

Class	${\mathrm{Sn}}_{c}$	${\mathrm{Sp}}_{c}$	${\mathrm{Pr}}_{c}$	$F1_{c}$	${\mathrm{ACC}}_{c}$
W	80.5 (1.5)	96.4 (0.4)	87.9 (1.2)	84.0 (0.9)	92.5 (0.4)
N1	63.1 (2.1)	89.0 (0.7)	50.5 (1.9)	56.1 (1.9)	85.1 (0.7)
N2	80.6 (1.0)	89.4 (0.5)	84.9 (0.7)	82.7 (0.8)	85.7 (0.6)
N3	79.2 (2.2)	97.9 (0.2)	74.2 (1.9)	76.6 (1.4)	96.5 (0.2)
REM	81.5 (2.0)	97.8 (0.2)	82.0 (1.5)	81.7 (1.4)	96.1 (0.3)

**Table 8:** Comparison between the proposed method and other end-to-end deep learning methods for automatic detection of sleep stages using a single EEG channel.

Study	Subjects	Input Signal	Classification Scheme	Class Imbalance Handling	DL Model	Overall Performance			Per Class Sn (${\mathrm{Sn}}_{c}$)				
						ACC	MF1	Sn	W	N1	N2	N3	REM
[2]	42	Fpz-Cz	One-to-One	Not Reported	Pre-trained CNN	83.6	-	74.4	92.8	28.3	93.3	71.6	84.0
[2]	42	Fpz-Cz	Many-to-One	Not Reported	Pre-trained CNN	84.7	-	76.7	94.5	36.0	92.7	72.9	86.9
[27]	20	Fpz-Cz	Many-to-One	Oversampling	CNN + RNN	82.0	76.9	78.7	83.4	50.1	81.7	94.2	83.9
[27]	20	Pz-Oz	Many-to-One	Oversampling	CNN + RNN	79.8	73.1	-	-	-	-	-	-
[36]	20	Fpz-Cz	Many-to-One	Subsampling	CNN	81.9	73.8	73.9	-	-	-	-	-
[10]	20	Fpz-Cz	Many-to-One	Subsampling	CNN	75.0	70.0	73.6	70.0	60.0	73.0	91.0	74.0
[37]	5728	C4-A1	Many-to-One	Subsampling	CNN	87.0	78.0	77.2	91.0	35.0	89.0	85.0	86.0
[5]	20	Fpz-Cz	Many-to-Many	Original Sampling	CNN + RNN	84.3	79.7	81.1	90.6	54.5	82.7	88.9	88.7
This Study	81	C4-M1	One-to-One	Original Sampling	Pre-trained CNN	75.4	70.0	68.8	85.8	37.9	85.9	65.5	69.1
This Study	81	C4-M1	One-to-One	Original Sampling	Pre-trained CNN + RNN	77.3	73.0	72.0	85.3	44.3	86.2	68.9	75.4
This Study	81	C4-M1	One-to-One	Oversampling	Pre-tained CNN + RNN	76.9	75.7	77.1	79.0	69.5	77.0	80.4	79.4

## Data Availability

The data sets that support the findings of this study are available from the Interdisciplinary Center of Sleep Medicine in Charité–Universitätsmedizin Berlin in Berlin, Germany and the Sleep Disorders Center in the University of Michigan in Ann Arbor, Michigan, USA. Restrictions apply to the availability of these data sets, which were used under license for the current study, and so are not publicly available. Both data sets can be made available upon approval of a research request by the Institutional Ethics Committee at Charité and the Institutional Review Board at the University of Michigan.
